# Impact of the PI-QUAL MRI quality score on histopathological up-staging from MRI fusion biopsy to final prostatectomy specimen

**DOI:** 10.1007/s00345-025-05755-6

**Published:** 2025-06-30

**Authors:** Maximilian Haack, Mohamad Turkman, Tobias Jorg, Lukas Müller, Gregor Duwe, Lisa Johanna Frey, Maximilian Peter Brandt, Axel Haferkamp, Hendrik Borgmann, Robert Dotzauer

**Affiliations:** 1 Department of Urology and Pediatric Urology, Johannes Gutenberg University Medical Center, Langenbeckstraße 1, 55131 Mainz, Rhineland-Palatinate Germany; 2 Department of Diagnostic and Interventional Radiology, Johannes Gutenberg University Medical Center, Langenbeckstraße 1, 55131 Mainz, Rhineland-Palatinate Germany; 3 Department of Urology, Faculty of Health Sciences, Theodor Fontane Medical School, Hochstraße 29, 14770 Brandenburg an der Havel, Germany

**Keywords:** PI-QUAL, Multiparametric MRI, Prostate cancer, Upstaging

## Abstract

**Purpose:**

The qualitative heterogeneity of multiparametric MRI (mpMRI) poses significant challenges for the diagnostic pathway of prostate cancer (PCa). The Prostate Imaging Quality Score (PI-QUAL) is a novel tool for the qualitative assessment of mpMRI. Aim of this study was to evaluate the impact of PI-QUAL on consistency of radiological to pathological T-stage.

**Methods:**

Patients undergoing MRI-TRUS fusion biopsy and radical prostatectomy (RP) from 01/2016 to 03/2024 were retrospectively included. PI-QUAL was determined by two expert radiologists and categorised: inadequate (1–2), sufficient (3) and optimal (4–5). Primary endpoint was upstaging from locally confined disease in mpMRI (mrT ≤ 2) to advanced in RP-specimen (pT ≥ 3a). Variables were compared using analysis of variance and χ^2^ or Fisher’s exact test. Uni- and multivariate binary regression identified independent predictors.

**Results:**

Of 349 patients included, 18 had PI-QUAL 1–2, 44 PI-QUAL 3 and 287 PI-QUAL 4–5. Patient characteristics, PI-RADS scores and biopsy counts were balanced between these groups. Upstaging from mrT ≤ 2 to pT ≥ 3a was significantly more frequent in PI-QUAL 1–2 (22.4%) and PI-QUAL 3 (22.7%) compared to PI-QUAL 4–5 (10.8%) (*p* = 0.031). Suboptimal mpMRI harbours an increased risk of upstaging (HR 2.22; 95% CI 1.05–4.71; *p* = 0.037). Optimal mpMRI quality independently predicts higher PI-RADS grading (PI-RADS ≥ 4) (HR 2.27; 95% CI 1.02-5.00; *p* = 0.043).

**Conclusion:**

PI-QUAL (≤ 3) significantly influences PI-RADS grading and predicts for upstaging from radiological to pathological staging. In case of suboptimal image quality, repetition of mpMRI should be considered.

**Supplementary Information:**

The online version contains supplementary material available at 10.1007/s00345-025-05755-6.

## Introduction

Multiparametric magnetic resonance imaging (mpMRI) of the prostate is considered the gold standard diagnostic method for targeted prostate biopsy [1–4]. The Prostate Imaging Reporting and Data System (PI-RADS) has been established to assess the possible malignancy of abnormal areas on mpMRI [5–7]. Since PI-RADS staging is used as one of the central parameters for deciding whether to perform a targeted prostate biopsy, a good quality mpMRI is essential. However, the quality of mpMRI can vary widely, which may affect the ability to assess the PI-RADS score [8–10]. This also may limit the detection power of MRI-TRUS fusion biopsy and expose patients to unnecessary morbidity due to poor initial diagnosis. In addition, mpMRI is used to assess the extent of the tumour, which is crucial information for preoperative planning regarding surgical margins, nerve preservation and bladder neck preparation to avoid compromising oncologic cure and urogenital function. However, accurate assessment of tumour extent remains a major challenge and is reflected in the wide variability in radiological staging [11, 12]. This is further exacerbated by the variable quality of mpMRI, resulting in a poor diagnostic starting point prior to radical prostatectomy (RP) or radiotherapy.

In order to evaluate and standardise the diagnostic consistency and quality of mpMRI examinations, Giganti et al. therefore developed a novel qualitative scoring system [13, 14]. The Prostate Imaging Quality (PI-QUAL) score is based on a 5-point Likert scale with PI-QUAL 1–2 defining mpMRI of non-diagnostic, PI-QUAL 3 sufficient and PI-QUAL 4–5 optimal diagnostic quality [13]. The PI-QUAL score complements PI-RADS criteria and ensures that image data meet the technical requirements and diagnostic standards necessary for reliable evaluation. However, only a few studies have investigated the influence of PI-QUAL on the agreement between radiological and pathological tumour stage [15, 16]. Therefore, the aim of this study was to further investigate the influence of the PI-QUAL score on the concordance between radiological and pathological tumour stage in a monocentric study design with standardised PI-QUAL assessment and surgical approach.

## Materials and methods

### Study design and population

**Fig. 1:**
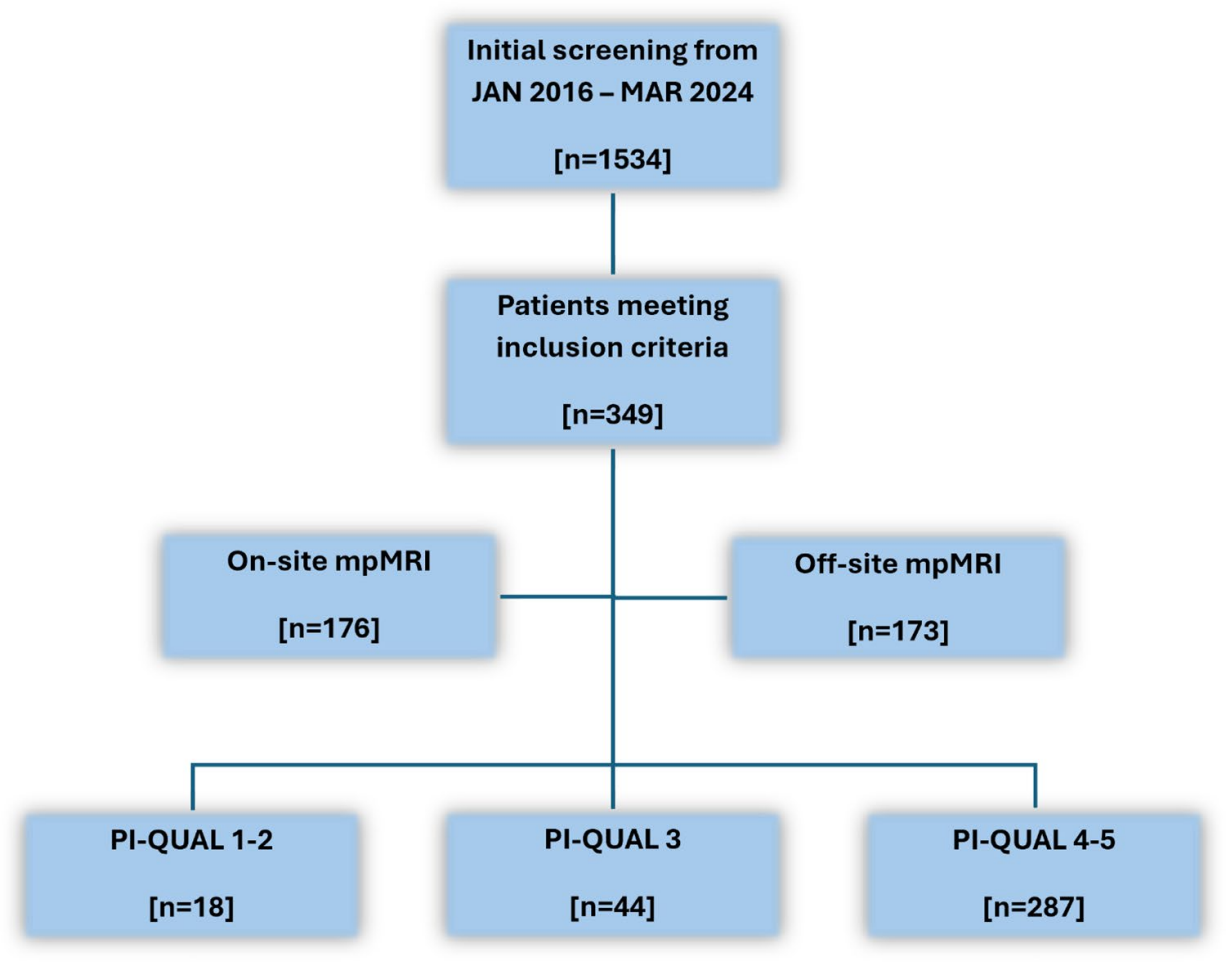
Flowchart of the study population and the corresponding sample numbers of the PI-QUAL groups

For this observational study, we retrospectively screened patients which underwent MRI-TRUS fusion biopsy as well as subsequent RP in case of proven prostate cancer from January 1st 2016 until March 30th 2024 at the University Medical Centre Mainz. Only patients who had a mpMRI of the prostate no more than 6 months before RP were included. Patients who previously had hormone therapy, any prostate surgery or radiotherapy were excluded. After initial screening, 349 patients were selected for further investigation (Fig. 1). The study protocol was reviewed and approved by the ethics committee of the state medical association of Rhineland-Palatinate, approval number 2024-17596-retrospective. Data processing was carried out in accordance with the applicable guidelines of the Rhineland-Palatinate State Hospital Act (§ 36, 37).

### Radiological requirements and PI-QUAL assessment

On- or off-site mpMRI which matched the following criteria, were included:

- Sequences: T1-weighted imaging (T1WI), T2-weighted imaging (T2WI), diffusion weighted imaging (DWI) and Dynamic contrast-enhanced MR imaging (DCE-MRI).
- PI-RADS classification 2.0 (before 2019) or 2.1 (after 2019), according to the latest version.
- Assessment of the radiological T-stage according to the European Society of Urogenital Radiology/EAU Section of Urologic Imaging consensus for expert radiologists [17].

The PI-QUAL score was then prospectively evaluated by two expert radiologists with a high diagnostic rate for mpMRI (> 1000 mpMRI reads overall and > 200 mpMRI reads/year [17]) and over 5 years of experience with prostate imaging. In case of discrepancies in the PI-QUAL results, the case was discussed, and a final score was determined. Both radiologists were blinded to the final pathological report after RP. The PI-QUAL scores were categorised into three groups of diagnostic value in concordance with the PRECISION trial by Giganti et al. [13]. PI-QUAL 1 and 2 were therefore considered diagnostically inadequate, PI-QUAL 3 sufficient and PI-QUAL 4 and 5 optimal. At the time of the evaluation, PI-QUAL version 1 was in use.

### MRI-TRUS fusion biopsy and surgical approach

MRI-TRUS fusion biopsy was performed under local anaesthesia and perioperative antibiotic prophylaxis. The targeted biopsies (2–4 per target) were taken from the suspicious lesion and a systematic 12-fold biopsy was also performed, with 6 biopsies from each side and two (1x medial and 1x lateral) at three levels (basal, mid and apical). Regarding surgical approach of RP, all 349 patients undergone robot-assisted, laparoscopic surgery.

### Primary and secondary outcomes

The primary endpoint was defined as the upstaging rate from radiologic T-status (mrT 2) to pathologic T-stage (pT ≥ 3a).

Differentiation to mrT 3 stage was based on any evidence of extraprostatic extension (EPE): neurovascular bundle asymmetry, irregular/speculated margin, capsular bulging/disruption, obliteration of the rectoprostatic angle, periprostatic fat infiltration [18, 19].

Secondary endpoints were the distribution of PI-QUAL scores between on-site and off-site radiology centers, relationship of PI-QUAL to PI-RADS score and the impact of PI-QUAL score on positive surgical margin (PSM) and vesicourethral leakage (VUL), as important surgical outcomes.

### Data analysis

For statistical analysis, χ^2^ and Fisher’s Exact Test were used for categorical variables. Continuous variables the analysed using students t-test or one-way ANOVA with Tukey’s multiple comparisons test. Univariate and multivariate binary logistic regression analysis were performed to evaluate independent predictors for primary and secondary endpoints. The level of significance was set at *p* < 0.05.

## Results

### Baseline characteristics

Overall, 349 patients were included in the study with a median age of 66 years (IQR 61–72), median iPSA of 7.9 ng/ml (IQR 5.9–12) and a median prostate volume of 45 ml (IQR 33-64.8). The most common clinical stage in the digito-rectal examination was cT1 (63.9%), followed by cT2 (27.8%) and cT3 (5.7%). The median number of biopsies was 16 (IQR 15.8–19), of which a median of 6 (IQR 3–9) were positive. The median number of targeted biopsies was 3 (IQR 3–4), of which a median of 2 (IQR 1–3) were positive. Of all patients included, 18 had inadequate (PI-QUAL 1–2), 44 had sufficient (PI-QUAL 3) and 287 had optimal mpMRI quality (PI-QUAL 4–5). Basic patient characteristics, serological parameters and biopsy counts (absolute and positive) were equally balanced between these groups (Table 1).

**Table 1:** Preoperative patient characteristics of the study population divided in three PI-QUAL groups

Preoperative patient characteristics	PI-QUAL 1–2 (*n* = 18)	PI-QUAL 3 (*n* = 44)	PI-QUAL 4–5 (*n* = 287)	*p*-value
Age, Median [a] (IQR)	68 (62–71)	66 (63–71)	66 (61–72)	0.474
Body Mass Index, Median [kg/m^2^] (IQR)	27,1 (25.7–32.1)	28.4 (24.4–30.8)	26.9 (24.8–29.4)	0.254
International Prostate Symptom Score, Median [n] (IQR)	7 (3.5–12.5)	4 (2-10.5)	8 (4–14)	0.227
International Index of Erectile Function Score, Median [n] (IQR)	12,5 (9.5–21.3)	17 (10-21.8)	17 (10–21)	0.778
American Society of Anesthesiologists Score (ASA), Median [n] (%)				0.226
ASA I	2 (11.1)	3 (6.8)	11 (3.8)	
ASA II	9 (50)	25 (56.8)	198 (70)	
ASA III	7 (38.9)	13 (29.5)	70 (24.4)	
ASA IV	0 (0)	0 (0)	1 (0,3)	
Missing	0 (0)	3 (6.8)	7 (2.4)	
Initial PSA, Median [ng/ml] (IQR)	6,0 (5.2–7.2)	7,5 (6.1–10.8)	8,1 (6.0–13.0)	0.170
Prostate volume, Median [ml] (IQR)	56.5 (29.5–78.8)	40 (35–60)	45 (33–65)	0.230
PSA density, Median [ng/ml/ml] (IQR)	0.12 (0.08–0.20)	0.17 (0.11–0.28)	0.19 (0.12–0.29)	0.232
Clinical T stage, [n] (%)				0.213
cT1	15 (83.3)	28 (63.6)	180 (62.7)	
cT2	2 (11.1)	10 (22.7)	85 (29.6)	
cT3	1 (5.6)	5 (11.4)	13 (4.5)	
cT4	0 (0)	0 (0)	1 (0.3)	
Missing	0 (0)	1 (2.3)	8 (2.8)	
Biopsy counts absolute, Median [n] (IQR)	16 (12–20)	18 (15.3–21)	16 (16–19)	0.237
Biopsy counts positive, Median [n] (IQR)	5 (3-7.5)	6 (3-9.8)	6 (3–9)	0.437
Targeted biopsy counts absolute, Median [n] (IQR)	3 (2–4)	3 (2–4)	3 (3–4)	0.065
Targeted biopsy counts positive, Median [n] (IQR)	2 (0-3.25)	2 (0–3)	2 (1–3)	0.398
D’Amico classification, n [%]				0.248
Low	6 (33.3)	5 (11.4)	57 (19.9)	
Intermediate	8 (44.4)	28 (63.6)	142 (49.7)	
High	4 (22.2)	11 (25.0)	87 (30.4)	

Continuous variables are presented as median and interquartile range and were tested by one-way ANOVA with tukey’s multiple comparisons test

Categorical variables were tested using χ^2^ or fisher’s exact test

*P* < 0.05 was considered significant

### Radiological parameters

The mpMRI was performed equally by on-site and off-site radiology centres (*n* = 176 vs. *n* = 173). Diagnostic quality from the off-site radiology centres was significantly inferior compared to the on-site radiology centre (Table 2). Interestingly, the on-site mpMRIs showed lower PI-RADS scores significantly more frequent than off-site mpMRI (*p* = 0.036): PI-RADS 1–2 (3% vs. 0%), PI-RADS 3 (11.8% vs. 8.1%), PI-RADS 4 (40.8% vs. 51.6%) and PI-RADS 5 (44.4% vs. 40.4%). Target lesions were predominantly located in the peripheral zone (68.8%) compared to the transitional zone (16.3%) or both (11.2%). However, there was no significant difference between the three PI-QUAL groups (*p* = 0.279). Reports of target location was missing in 3.7% of cases. Regarding mrT and mrN stage, there was no significant difference between the three PI-QUAL groups (Table 2). Radiological nodal staging was missing in 7.2% cases.

**Table 2:** Radiological parameters of the study population divided in three PI-QUAL groups

Radiological parameters	PI-QUAL 1–2 (*n* = 18)	PI-QUAL 3 (*n* = 44)	PI-QUAL 4–5 (*n* = 287)	*p*-value
Radiological center, n (%)				< 0.001
On-site (*n* = 176)	5 (2.8)	12 (6.8)	159 (90.3)	
Off-site (*n* = 173)	13 (7.5)	32 (18.5)	128 (74.0)	
PI-RADS, n (%)				0.284
< 3	1 (5.6)	2 (4.5)	6 (2.1)	
3	3 (16.7)	5 (11.4)	25 (8.7)	
4	9 (50.0)	18 (40.9)	125 (43.5)	
5	4 (22.2)	15 (34.1)	121 (42.2)	
Missing	1 (5.6)	4 (9.1)	10 (3.5)	
ROI-location, n (%)				0.279
Transitional zone	4 (22.2)	11 (25.0)	42 (14.6)	
Peripheral zone	12 (66.7)	27 (61.4)	201 (70.0)	
Both	1 (5.6)	3 (6.8)	35 (12.2)	
Missing	1 (5.6)	3 (6.8)	9 (3.1)	
MRI staging				
*T stage*,* n (%)*				0.314
mrT2	13 (72.2)	38 (86.4)	193 (67.2)	
mrT3a	4 (22.2)	4 (9.1)	55 (19.2)	
mrT3b	1 (5.6)	2 (4.5)	28 (9.8)	
mrT4	0 (0)	0 (0)	11 (3.8)	
*N stage*,* n (%)*				0.661
mrN0	17 (94.4)	36 (81.8)	249 (86.8)	
mrN1	0 (0)	3 (6.8)	19 (6.6)	
Missing	1 (5.6)	5 (11.4)	19 (6.6)	

Categorical variables were tested using χ^2^ or fisher’s exact test

*P* < 0.05 was considered significant

### Pathological parameters

In group comparison, there was no significant difference between the three PI-QUAL groups in terms of final International Society of Urological Pathology (ISUP) grade, pT stage and pN stage (Table 3). Lymph node staging was not performed in 72 cases since preoperative staging using D’Amico and National Comprehensive Cancer Network (NCCN) classification [20, 21] predicted a low to intermediate risk of advanced disease. Regarding the surgical margins of the final RP-specimen, there was a trend towards more frequent PSM in the worst PI-QUAL group 1–2 (38.9% vs. 31.8% vs. 28.2%; *p* = 0.553) which however was not significantly different (Table 3).

**Table 3:** Pathological parameters of the study population divided in three PI-QUAL groups

Pathological parameters	PI-QUAL 1–2 (*n* = 18)	PI-QUAL 3 (*n* = 44)	PI-QUAL 4–5 (*n* = 287)	*p*-value
ISUP grade, n (%)				0.283
1	2 (11.1)	3 (6.8)	32 (11.1)	
2	11 (61.1)	28 (63.6)	162 (56.4)	
3	1 (5.6)	10 (22.7)	62 (21.6)	
4	2 (11.1)	0 (0)	7 (2.4)	
5	2 (11.1)	3 (6.8)	24 (8.4)	
Histopathological staging				
*T stage*,* n (%)*				0.537
pT2	13 (72.2)	29 (65.9)	192 (66.9)	
pT3a	5 (27.8)	8 (18.2)	54 (18.8)	
pT3b	0 (0)	7 (15.9)	40 (13.9)	
pT4	0 (0)	0 (0)	1 (0.4)	
*N stage*,* n (%)*				0.395
pN0	10 (55.6)	32 (72.7)	195 (67.9)	
pN1	1 (5.5)	5 (11.4)	34 (11.9)	
Missing	7 (38.9)	7 (15.9)	58 (20.2)	
Positive surgical margin, n (%)	7 (38.9)	14 (31.8)	81 (28.2)	0.553

Categorical variables were tested using χ^2^ or fisher’s exact test

*P* < 0.05 was considered significant

### Upstaging rates

In over 75% of patients with sufficient (PI-QUAL 3) and optimal (PI-QUAL 4–5) mpMRI quality, the radiological T stage was consistent with the final pathological T stage (as defined above), whereas this was only the case in about half of the patients with insufficient (PI-QUAL 1–2) mpMRI quality (Table 4). In contrast, upstaging in the final RP-specimen (pT ≥ 3a) was significantly more frequent in the PI-QUAL 1–2 and even PI-QUAL 3 group compared to PI-QUAL 4–5 (22.2% vs. 22.7% vs. 10.8%; *p* = 0.031). There was also a significant increase in downstaging in the PI-QUAL 1–2 group compared to PI-QUAL 4–5 (22.2% vs. 10.8%; *p* = 0.040). Interestingly, upstaging was significantly more frequent in cases where the target lesion was located in the transition zone compared to peripheral zone or both zones (24.6% vs. 10.4% vs. 10.3%; *p* = 0.023). However, mpMRIs that showed transitional zone target lesions were also more likely to be of suboptimal quality (27.8% vs. 17.3%; *p* = 0.087) (Table 2).

**Table 4:** Comparison of radiological tumour stage (mrT) with final pathological tumour stage (pT) between the three PI-QUAL groups

Comparison mrT vs. pT stage, *n* (%)	PI-QUAL 1–2 (*n* = 18)	PI-QUAL 3 (*n* = 44)	PI-QUAL 4–5 (*n* = 287)	*p*-value
Agreement	10 (55.6)	33 (75)	225 (78.4)	0.084
Upstaging	**4 (22.2)**	**10 (22.7)**	**31 (10.8)**	**0.031**
Downstaging	4 (22.2)	1 (2.3)	31 (10.8)	0.040

Upstaging was definded as mrT2 to pT ≥ 3a. The other endpoints agreement and downstaging were also defined on the basis of this distinction. Categorical variables were tested using fisher’s exact test

*P* < 0.05 was considered significant

### Risk factor analysis

In multivariate binary logistic regression analysis, suboptimal image quality (PI-QUAL ≤ 3) was an independent predictor for upstaging from mrT-stage (≤ 2) to pT-stage in the RP-specimen (≥ 3a) (HR 2.22; 95% CI 1.05–4.71; *p* = 0.037). Interestingly, a suspicious digitorectal examination (cT ≥ 2) tended to be an independent predictor of pathological agreement/downstaging compared to former mrT-stage (HR 0.49; 95% CI 0.22–1.09; *p* = 0.079). Other variables did not prove to be independent predictors of this endpoint (Table 5). Regarding PI-RADS, we found that optimal mpMRI quality (PI-QUAL ≥ 4) (HR 2.27; 95% CI 1.02-5.00; *p* = 0.043) and clinical T-stage ≥ 2 (HR 2.77; 95% CI 1.17–6.54; *p* = 0.020) were independent predictors of a high PI-RADS score (≥ 4) (Table 5). For PSM, higher iPSA (HR 1.04; 95% CI 1.01–1.07; *p* = 0.004) and a higher positive biopsy count (HR 1.11; 95% CI 1.04–1.19; *p* = 0.002) proved to be independent predictors (Table 5). VUL was significantly predicted by higher age (HR 1.10; 95% CI 1.02–1.19; *p* = 0.016) and prostate volume (HR 1.01; 95% CI 1.00-1.02; *p* = 0.038). Interestingly, preservation of erectile nerves showed to be a negative predictor of VUL (HR 0.24; 95% CI 0.07–0.88; *p* = 0.031) (Table 5).

With regard to factors influencing the quality of the mpMRI itself, multivariate binary logistic regression analysis confirmed that off-site mpMRI was an independent predictor of a suboptimal PI-QUAL score (≤ 3) (HR 3.57; 95% CI 1.89–6.67; *p* < 0.001) (Supplementary Table 1). Other variables did not prove to be independent predictors of PI-QUAL ≤ 3.

**Table 5:** Multivariate binary logistic regression for upstaging from radiologic T-status (mrT 2) to pathologic T-stage (pT ≥ 3a); PI-RADS ≥ 4; positive surgical margin (PSM) and vesicourethral leakage (VUL)

	Upstaging			PI-RADS ≥ 4			PSM			VUL		
	HR	95% CI	p-value	HR	95% CI	p-value	HR	95% CI	p-value	HR	95% CI	p-value
**PI-QUAL ≤ 3**	**2.22**	**1.05–4.71**	**0.037**	**0.44**	**0.20–0.98**	**0.043**	1.34	0.69–2.62	0.392	1.85	0.66–5.19	0.240
Offsite	1.43	0.72–2.85	0.312	1.91	0.96–3.81	0.066	1.47	0.85–2.54	0.164	1.00	0.40–2.48	0.996
Age	0.97	0.92–1.01	0.145	1.02	0.97–1.07	0.487	1.00	0.96–1.04	0.920	**1.10**	**1.02–1.19**	**0.016**
BMI	1.02	0.94–1.10	0.632	1.06	0.97–1.16	0.185	1.00	0.94–1.07	0.975	1.04	0.94–1.15	0.421
Prostate volume	1.00	0.99–1.01	0.985	1.00	0.99–1.02	0.642	1.00	0.99–1.01	0.512	**1.01**	**1.00-1.02**	**0.038**
iPSA	1.00	0.97–1.03	0.948	1.04	0.99–1.11	0.140	**1.04**	**1.01–1.07**	**0.004**	1.01	0.97–1.04	0.712
cT-stage ≥ 2	0.49	0.22–1.09	0.079	**2.77**	**1.17–6.54**	**0.020**	1.56	0.90–2.70	0.113	2.02	0.84–4.85	0.115
Positive biopsy count							**1.11**	**1.04–1.19**	**0.002**	1.00	0.89–1.12	0.989
Surgery time							1.00	1.00-1.01	0.133	1.00	1.00-1.01	0.181
Nerve sparing							0.94	0.51–1.71	0.828	**0.24**	**0.07–0.88**	**0.031**

Depicted are hazard ratio (HR), 95% confidence interval (CI)

*P* < 0.05 was considered significant

## Discussion

Our results provide important evidence that the quality of mpMRI has a direct impact on assessability of PI-RADS and tumour extent. As a novel objective and standardised evaluation tool for mpMRI quality, the PI-QUAL score thus plays an increasingly important role in the diagnostic cascade of prostate cancer. We were able to show that upstaging from organ-confined (mrT 2) to advanced disease in final RP-specimen (pT ≥ 3a) as well as low PI-RADS scores (≤ 3) were significantly predicted by suboptimal mpMRI quality (PI-QUAL ≤ 3). In addition, we found that the quality of the mpMRIs from decentralised radiology centres was significantly worse. Therefore, patients with high clinical suspicion of advanced prostate cancer (e.g. cT ≥ 2) and mpMRI from decentralised radiology centres should at least have the mpMRI re-assessed by experienced radiologists or undergo repeat mpMRI of high quality (PI-QUAL ≥ 4) to avoid the risk of upstaging and the associated poorer preoperative preparation and planning. Our data thus support the results of the study by Windisch et al., who also found an independent association of inadequate mpMRI quality (PI-QUAL 1–2) with an increased upstaging rate in a very similar patient population (*n* = 351 compared to ours *n* = 349) [15]. However, in our patient population this effect was already evident at a PI-QUAL score of 3 and below. Furthermore, due to multiple investigators evaluating the PI-QUAL score and non-standardised surgical techniques of RP, their study cohort was rather inhomogeneous. This observation is supported by the results of Dinneen et al., who found an increased rate of EPE in the final RP-specimen in patients with inadequate to sufficient mpMRI quality (PI-QUAL 1–3), although only organ-confined tumour extension was described on the corresponding mpMRI [16]. PI-QUAL 1–3 was also an independent predictor for EPE in multivariate regression analysis [16]. As EPE has been described as an indicator for a pT3a stage [18, 22–25], which was the cut-off for upstaging for our primary endpoint, these results support our own observations even further. The fact that the colleagues were also able to show increased upstaging from PI-QUAL ≤ 3 and not only from PI-QUAL 1–2 contrasts the results of Windisch et al. and emphasises the importance of optimal mpMRI quality.

This is particularly interesting as the founders of this qualitative assessment tool for mpMRI considered a PI-QUAL score of 3 to be of sufficient quality to rule in but not to rule out all significant lesions [13, 14]. However, our data raise the question of whether a PI-QUAL of 3 is actually suitable for predicting significant lesions, as it was associated with a significantly lower detection of PI-RADS ≥ 4 in our large cohort. It should also be noted that the PI-QUAL score was not validated on the final pathological tumour stage at RP, but on the pathological results of the MRI-TRUS fusion biopsy [13]. With only about 50% concordance between the pathological results of the MRI-TRUS fusion biopsy and RP, the endpoint for validation of the PI-QUAL score in the diagnostic cascade is probably too early [26, 27]. Therefore, the results of our study and those of Windisch et al. [15] and Dinneen et al. [16] provide important evidence that the PI-QUAL score should be validated against the final pathological tumour stage in addition to the biopsy result. In contrast to the multicentre patient cohort of Windisch et al., the patients in our study all underwent biopsy and surgery at our clinic with the same surgical approach. In addition, the PI-QUAL assessment has been carried out in duplicate by two expert radiologists, which provides an additional level of standardisation. As these are the only studies to our knowledge, that investigated tumour staging across all diagnostic steps, further research is desperately needed to confirm these findings.

We also investigated other risk factors, such as BMI, prostate volume, iPSA and clinical T-stage, which could impair diagnostic quality. However, in line with the findings of Pausch et al., there was no significant correlation between these parameters with lower PI-QUAL scores [28]. As already described in other studies, factors such as slice thickness, patient movement, bowel peristalsis, rectal distension and medical implants play a much more decisive role for good image quality in mpMRI [29, 30].

Regarding the influence of target localisation on mpMRI, our study population surprisingly showed an increased upstaging rate from target lesions located in the transitional zone. Contrary to the expectation that an increased rate of extra-prostatic tumour growth (≥ pT3a) would be more likely to occur in the peripheral zone, Wu et al. also demonstrated an increased risk of upstaging in the final RP-specimen in transitional zone target lesions compared to peripheral lesions [31]. In line with other studies, they attributed this observation to the presence of perineural infiltration, which allows the tumour to more easily pass through the capsule along the nerve fibres [31–33]. Furthermore, Chen et al. identified target lesions located in the transition zone (particularly the anterior transition zone) as an independent predictor of ISUP upstaging in the final RP-specimen [34]. As the MRI in this subgroup more often showed PI-QUAL ≤ 3, it was reasonable to assume that the increased upstaging rate could be due to the suboptimal image quality. Therefore, we repeated the multivariate binary regression analysis and included target lesion location. This showed that it had no effect on the upstaging rate and actually increased the predictive power of the PI-QUAL score (HR 2.35; 95% CI 1.09–5.06; *p* = 0.029) (Supplementary Table 6).

As a further aspect of our work, we examined the influence of suboptimal mpMRI quality (PI-QUAL ≤ 3) on PSM and VUL, as important surgical outcomes. Although we could not find an independent correlation with the PI-QUAL score, an increased PSM was independently predicted by a higher iPSA and number of positive biopsies. This is not surprising, as a higher iPSA and increased positive biopsies indicate a more extensive tumour stage, and are therefore known risk factors for PSM [35–38]. Regarding the influence of PI-QUAL on VUL, we were able to show that higher prostate volume was an independent risk factor for the occurrence of VUL, which is in line with other studies [39–41]. However, PI-QUAL did not provide any predictive value for VUL.

Our study also has some limitations due to the retrospective study design. Whereas PI-QUAL scores were assessed independently by two expert radiologists for mpMRI, PI-RADS scores were defined by several radiologists with different levels of experience in different radiology centres. Therefore, PI-RADS scores were not measured in the same standardised way as PI-QUAL. However, as approx. 50% of mpMRI interpretations were carried out at our high-volume radiologic centre with standardised PI-RADS assessment and experienced validation, this is a relatively minor limitation. In addition, a subsequent change in the PI-RADS score would distort the treatment pathway and any treatment decisions based on it (e.g. the decision for nerve preservation). In a prospective study design, a standardised PI-RADS scoring would certainly be of great advantage, as all subsequent treatment steps could be based on it. Furthermore, the distribution of the three PI-QUAL groups was rather uneven, which limits the comparison. However, as the studies of Windisch et al. and Dinneen et al. show [15, 16], the diagnostic quality of mpMRI is already very good in large, industrialised countries. It will therefore be difficult to achieve an equally weighted group distribution. In addition, due to the study design we only investigated the effect of PI-QUAL in patients with proven prostate cancer. Also, PI-QUAL version 1 was still used, as this was the standard version at the time of the evaluation. Although comparing the radiological T stage with the final pathological T stage at RP has greater predictive power than only biopsy results, our data does not provide a negative control in patients without prostate cancer. However, as shown by Pausch et al., inadequate mpMRI quality also appears to be an independent predictor of an increased rate of clinically significant prostate cancer in patients with an initially false-negative MRI-TRUS fusion biopsy [28]. This underlines the importance of standardised quality assessment of mpMRI in the diagnostic cascade of prostate cancer.

## Conclusion

Our study is currently the largest single-center analysis of the impact of PI-QUAL across the entire diagnostic pathway of localised prostate cancer. We were able to show that suboptimal mpMRI quality (PI-QUAL ≤ 3) is the only independent predictor of upstaging to advanced tumour stage in the RP-specimen (pT ≥ 3a) and a strong independent predictor of lower PI-RADS grading (≤ 3). Furthermore, decentralized mpMRI is significantly more likely to be of suboptimal quality. However, we could not demonstrate an impact of PI-QUAL on PSM or VUL. To avoid underdiagnosis and poorer oncological outcomes in patients with localised prostate cancer, mpMRI of optimal quality (PI-QUAL ≥ 4) should be available before a treatment decision is made and should otherwise be repeated.

## Electronic supplementary material

Below is the link to the electronic supplementary material.

Supplementary Material 1

## Data Availability

Research data are not publicly available on legal or ethical grounds. Research data can be provided on request in pseudonymized form. Further enquiries can be directed to the corresponding author.
