# Supplementary material for: Impact of the PI-QUAL MRI quality score on histopathological up-staging from MRI fusion biopsy to final prostatectomy specimen
Source: World J Urol. 2025 Jun 30;43(1):404. doi: 10.1007/s00345-025-05755-6 (PMC12208975; doi:10.1007/s00345-025-05755-6)
Supplement: Supplementary file 1 — Supplementary Material 1 [file 345_2025_5755_MOESM1_ESM.docx]

**SUPPLEMENTARY MATERIALS**

**Supplementary Table 1**: Multivariate binary logistic regression for PI-QUAL ≥4. Depicted are hazard ratio (HR), lower and upper range of 95 % confidence interval (CI). P<0.05 was considered significant.

| PI-QUAL ≥4 | HR | Lower CI | Upper CI | p-value |
| --- | --- | --- | --- | --- |
| Off-site mpMRI | **0.26** | **0.14** | **0.51** | **<0.001** |
| Age | 0.97 | 0.93 | 1.01 | 0.180 |
| BMI | 0.95 | 0.88 | 1.02 | 0.126 |
| Prostate volume | 1.00 | 0.99 | 1.01 | 0.746 |
| iPSA | 1.02 | 0.97 | 1.06 | 0.441 |
| Clinical T-stage ≥2 | 1.27 | 0.65 | 2.51 | 0.488 |
| ROI in TZ | ref | ref | ref | 0.088 |
| ROI in PZ | 1.95 | 0.92 | 4.10 | 0.080 |
| ROI in TZ and PZ | **3.70** | **1.03** | **13.25** | **0.045** |

**Supplementary Table 2**: Univariate binary logistic regression for upstaging from radiologic T-status (mrT 2) to pathologic T-stage (pT ≥3a). Depicted are hazard ratio (HR) and 95 % confidence interval (CI). P<0.05 was considered significant.

| Upstaging | HR | 95% CI | p-value |
| --- | --- | --- | --- |
| PI-QUAL ≤3 | **2.41** | **1.19-4.86** | **0.014** |
| Offsite | 1.60 | 0.85-3.03 | 0.147 |
| Positive surgical margin | 0.79 | 0.39-1.64 | 0.532 |
| Nerve sparing | 1.40 | 0.75-2.63 | 0.296 |
| Biopsy count | 1.03 | 0.95-1.11 | 0.492 |
| Positive biopsy count | 0.98 | 0.91-1.06 | 0.611 |
| iPSA | 0.99 | 0.95-1.02 | 0.423 |
| Prostate volume | 1.00 | 0.99-1.01 | 0.677 |
| BMI | 1.04 | 0.97-1.12 | 0.307 |
| Age | 0.98 | 0.94-1.02 | 0.251 |
| ASA classification |  |  |  |
| *ASA I* | Reference |  |  |
| *ASA II* | 1.07 | 0.23-4.91 | 0.928 |
| *ASA III* | 1.70 | 0.36-8.14 | 0.507 |
| *ASA IV (only 1 patient)* | 0 | 0- | 1.000 |
| IIEF | 0.97 | 0.93-1.02 | 0.268 |
| IPSS | 0.99 | 0.94-1.04 | 0.629 |
| cT Stage ≥2 | **0.49** | **0.23-1.06** | **0.071** |
| PI-RADS ≥4 | 1.62 | 0.55-4.76 | 0.382 |
| D’Amico risk classification |  |  |  |
| *Low* | Reference |  |  |
| *Intermediate* | 0.77 | 0.35-1.68 | 0.509 |
| *High* | 0.63 | 0.26-1.54 | 0.308 |

**Supplementary Table 3:** Univariate binary logistic regression for PI-RADS ≥4. Depicted are hazard ratio (HR) and 95 % confidence interval (CI). P<0.05 was considered significant.

| PI-RADS ≥4 | HR | 95% CI | p-value |
| --- | --- | --- | --- |
| PI-QUAL ≤3 | **0.48** | **0.23-0.97** | **0.041** |
| Offsite | 0.69 | 0.37-1.29 | 0.239 |
| iPSA | 1.06 | 1.00-1.12 | 0.065 |
| Prostate volume | 1.00 | 1.00-1.02 | 0.466 |
| BMI | 1.05 | 0.96-1.14 | 0.272 |
| Age | 1.01 | 0.96-1.05 | 0.829 |
| cT Stage ≥2 | **2.96** | **1.28-6.88** | **0.011** |

**Supplementary Table 4:** Univariate binary logistic regression for positive surgical margin (PSM) Depicted are hazard ratio (HR) and 95 % confidence interval (CI). P<0.05 was considered significant.

| Positive surgical margin | HR | 95% CI | p-value |
| --- | --- | --- | --- |
| PI-QUAL ≤3 | 1.25 | 0.69-2.27 | 0.454 |
| Upstaging | 0.79 | 0.39-1.64 | 0.532 |
| Offsite | 1.30 | 0.82-2.07 | 0.271 |
| Nerve sparing | **0.57** | **0.35-0.94** | **0.027** |
| Biopsy count | 0.98 | 0.92-1.04 | 0.489 |
| Positive biopsy count | **1.13** | **1.07-1.19** | **<0.001** |
| iPSA | **1.05** | **1.03-1.08** | **<0.001** |
| Prostate volume | 1.00 | 0.99-1.00 | 0.290 |
| BMI | 1.01 | 0.95-1.07 | 0.755 |
| Age | 1.00 | 0.97-1.04 | 0.878 |
| Operation time | 1.01 | 1.00-1.01 | 0.020 |
| Insufficient VUA | **3.82** | **1.74-8.42** | **0.001** |
| Hospitalisation time | 1.05 | 0.98-1.12 | 0.146 |
| Post-OP cathererisation time | 1.06 | 1.00-1.12 | 0.047* |
| ASA classification |  |  |  |
| *ASA I* | Reference |  |  |
| *ASA II* | 7.16 | 0.94-54.70 | 0.058 |
| *ASA III* | **8.56** | **1.09-67.25** | **0.041** |
| *ASA IV (only 1 patient)* | 0 | 0- | 1.000 |
| IIEF | 0.98 | 0.95-1.02 | 0.326 |
| IPSS | 1.01 | 0.98-1.05 | 0.545 |
| cT Stage ≥2 | **0.50** | **0.30-0.82** | **0.006** |
| PI-RADS ≥4 | 1.53 | 0.73-3.22 | 0.264 |
| D’Amico risk classification |  |  |  |
| *Low* | Reference |  |  |
| *Intermediate* | 0.91 | 0.47-1.78 | 0.785 |
| *High* | 2.37 | 1.20-4.70 | 0.014 |

**Supplementary Table 5:** Univariate binary logistic regression for vesicourethral leakage (VUL). Depicted are hazard ratio (HR) and 95 % confidence interval (CI). P<0.05 was considered significant.

| Vesicourethral leakage | HR | 95% CI | p-value |
| --- | --- | --- | --- |
| PI-QUAL ≤3 | 1.61 | 0.65-3.98 | 0.300 |
| Upstaging | 0.50 | 0.11-2.17 | 0.353 |
| Offsite | 0.74 | 0.34-1.61 | 0.441 |
| Nerve sparing | **0.17** | **0.05-0.56** | **0.004** |
| Positive surgical margin | **3.82** | **1.74-8.42** | **0.001** |
| Biopsy count | 0.97 | 0.88-1.07 | 0.561 |
| Positive biopsy count | 1.00 | 0.92-1.10 | 0.923 |
| iPSA | 1.02 | 0.99-1.04 | 0.220 |
| Prostate volume | **1.02** | **1.01-1.03** | **<0.001** |
| BMI | 1.04 | 0.95-1.14 | 0.403 |
| Age | **1.12** | **1.05-1.20** | **0.001** |
| Operation time | 1.01 | 1.00-1.01 | 0.043* |
| Hospitalisation time | 1.26 | 1.11-1.42 | <0.001 |
| Post-OP cathererisation time | 1.24 | 1.13-1.36 | <0.001 |
| IIEF | 0.89 | 0.84-0.95 | <0.001 |
| IPSS | 1.05 | 0.99-1.11 | 0.078 |
| cT Stage ≥2 | 1.74 | 0.78-3.85 | 0.174 |
| PI-RADS ≥4 | 2.06 | 0.47-9.01 | 0.337 |
| D’Amico risk classification |  |  |  |
| *Low* | Reference |  |  |
| *Intermediate* | 2.39 | 0.52-10.95 | 0.263 |
| *High* | 5.25 | 1.15-23.90 | 0.032 |

**Supplementary Table 6:** Multivariate binary logistic regression for upstaging from radiologic T-status (mrT 2) to pathologic T-stage (pT ≥3a). Depicted are hazard ratio (HR) and 95 % confidence interval (CI). P<0.05 was considered significant.

|  | Upstaging | | |
| --- | --- | --- | --- |
|  | HR | 95% CI | p-value |
| PI-QUAL ≤3 | **2.35** | **1.09-5.06** | **0.029** |
| Offsite | 1.63 | 0.80-3.33 | 0.175 |
| Age | 0.96 | 0.92-1.01 | 0.130 |
| BMI | 1.01 | 0.93-1.10 | 0.786 |
| Prostate volume | 1.00 | 0.99-1.01 | 0.942 |
| iPSA | 1.00 | 0.97-1.04 | 0.805 |
| cT-stage ≥2 | 0.49 | 0.22-1.10 | 0.082 |
| ROI in TZ | Reference |  | 0.680 |
| ROI in PZ | 0.97 | 0.40-2.34 | 0.951 |
| ROI in TZ+PZ | 0.55 | 0.13-2.42 | 0.431 |
